# Methamphetamine Increases Locomotion and Dopamine Transporter Activity in Dopamine D5 Receptor-Deficient Mice

**DOI:** 10.1371/journal.pone.0075975

**Published:** 2013-10-14

**Authors:** Seiji Hayashizaki, Shinobu Hirai, Yumi Ito, Yoshiko Honda, Yosefu Arime, Ichiro Sora, Haruo Okado, Tohru Kodama, Masahiko Takada

**Affiliations:** 1 Tokyo Metropolitan Institute of Medical Science, Tokyo, Japan; 2 Japan Science and Technology Agency, CREST, Tokyo, Japan; 3 Department of Biological Psychiatry, Tohoku University Graduate School of Medicine, Sendai, Japan; 4 Systems Neuroscience Section, Primate Research Institute, Kyoto University, Inuyama, Japan; Nathan Kline Institute and New York University School of Medicine, United States of America

## Abstract

Dopamine regulates the psychomotor stimulant activities of amphetamine-like substances in the brain. The effects of dopamine are mediated through five known dopamine receptor subtypes in mammals. The functional relevance of D5 dopamine receptors in the central nervous system is not well understood. To determine the functional relevance of D5 dopamine receptors, we created D5 dopamine receptor-deficient mice and then used these mice to assess the roles of D5 dopamine receptors in the behavioral response to methamphetamine. Interestingly, D5 dopamine receptor-deficient mice displayed increased ambulation in response to methamphetamine. Furthermore, dopamine transporter threonine phosphorylation levels, which regulate amphetamine-induced dopamine release, were elevated in D5 dopamine receptor-deficient mice. The increase in methamphetamine-induced locomotor activity was eliminated by pretreatment with the dopamine transporter blocker GBR12909. Taken together, these results suggest that dopamine transporter activity and threonine phosphorylation levels are regulated by D5 dopamine receptors.

## Introduction

Dopamine mediates a wide variety of physiological and behavioral functions in the central nervous system (CNS), such as the response to psychomotor stimulants and reward and learning behaviors [1], [2], [3], [4], [5], [6], [7]. These roles of the dopamine system were discovered through the creation and characterization of dopamine receptor-deficient mice ([8], [9], [10], [11]. The effects of dopamine are mediated through five known subtypes of dopamine receptors in mammals (D1R, D2R, D3R, D4R, and D5R) [12].

Genomic studies found a significant relation between a polymorphism in the D5R gene locus and vulnerability to drug abuse [13], [14]. Consistent with this mutation, several studies found that D5Rs play a role in mediating the response to cocaine administration. D5R-deficient mice with a mixed genetic background are less sensitive to acute cocaine administration than control littermates [15]. Furthermore, D5R-deficient mice with a C57/B6 background are more sensitive to chronic cocaine administration than wild-type (WT) littermates [16]. However, it is unknown whether D5Rs contribute to the response to amphetamine-like drugs. To this end, we investigated the effect of D5R deficiency on methamphetamine (METH)-induced behavior. METH is a derivative of amphetamine and is a major psychostimulant that is frequently abused.

We found that D5R-deficient mice were hypersensitive to acute METH challenges. We also found that GBR12909, a dopamine transporter (DAT) blocker, affected the blocking and reversal of monoamine reuptake by METH through monoamine transporters such as DAT. In addition, we evaluated threonine phosphorylation levels in WT and D5R-KO mice because a specific threonine residue in DAT is important for modification of reuptake and release of dopamine [17], [18], [19], and found that threonine phosphorylation levels were higher in D5R-KO mice than in WT mice. Finally, we measured dopamine levels in the nucleus accumbens (NA) to assess whether this brain region mediated the altered hypersensitivity to METH but failed to detect a significant difference in dopamine levels in this brain region between WT and D5R-KO mice.

## Results

### Characterization of D5R-KO mice

**Figure 1:**
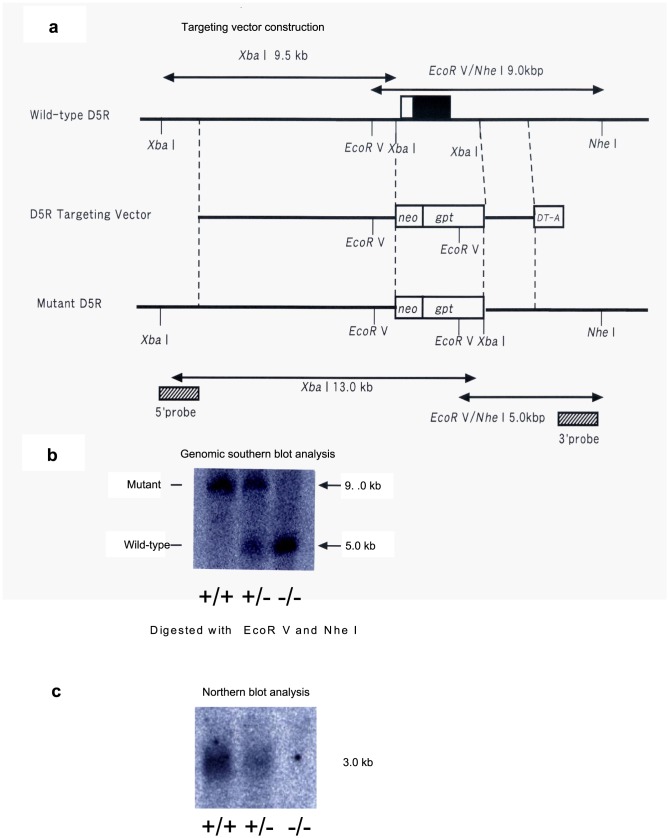
Generation of D5R-KO mice. (a) Design of the D5R gene targeting vector. Upper diagram: restriction enzyme map for the WT D5R gene locus. The black part of the box corresponds to the D5R gene coding region and the white part of the box represents the noncoding region. Middle diagram: the D5R gene targeting vector. Lower diagram: the D5R gene locus in the D5R-KO mice. Bottom diagram: Probes used for recombinant ES cell screening are indicated. (b) Genomic Southern blotting with a 3′ region probe. Genomic DNA was collected from WT (+/+), heterogeneous (+/−), and homogenous (−/−) D5R mice and subjected to electrophoresis and Southern blotting. The bands corresponding to wild-type and mutant DNA are indicated. (c) mRNA was collected from WT (+/+), heterogeneous (+/−), and homogenous (−/−) animals and subjected to electrophoresis and Northern blotting with a D5R cDNA probe. D5R mRNA was absent from the homogenous (−/−) D5R-KO animals.

We created a D5R-KO mice line on a C57/B6 background for this study. The murine D5R gene was disrupted in embryonic stem (ES) cells by homologous recombination that resulted in inactivation of the coding region (Figure 1a). Consistent with a previous study, the D5R-KO mice were fertile [20]. The authenticity of the D5R-KO line was confirmed by genomic Southern blotting with a 3′ region probe (Figure 1b). In addition, Northern blotting showed that D5R mRNA was completely abolished in the D5R-KO mice (Figure 1c).

### Effects of pharmacological manipulations on ambulation

To assess the roles of D5Rs in dopamine-mediated behaviors, we measured open field locomotor activities of WT and D5R-KO mice that were administered 2.5 mg/kg of METH via intraperitoneal injections. METH affects dopamine transmission by blocking dopamine reuptake and reversing dopamine release through the DAT pore. Therefore, we also evaluated the METH-induced locomotor activities after pretreatments with either saline or the DAT blocker GBR12909. Three-way analysis of variance (ANOVA) was employed to analyze METH challenge-induced locomotor activity data from the four groups of mice. The analysis was performed based on the following three factors: 1) pretreatment with saline control or GBR12909; 2) genotype (WT or D5R-KO); and 3) time course.

**Figure 2:**
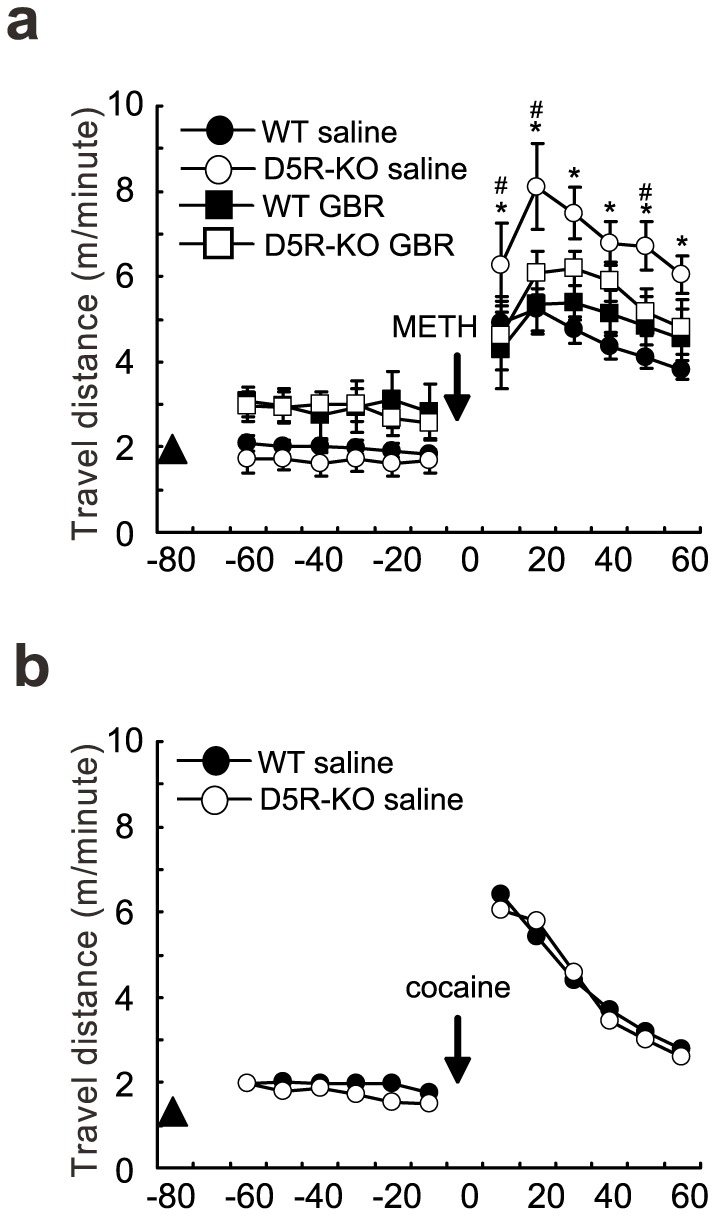
Increased METH-induced ambulatory activity in D5R-KO mice. (a) Open field locomotor activity after challenge with METH (2.5 mg/kg). Locomotion was measured for 60 min following each injection. The data were presented in 10 min time bins. The circles or squares represent the mean and the error bars represent the s.e.m. The pretreatments (arrowhead) were either saline (WT, filled circles, *n* = 6; and D5R-KO, open circles, *n* = 6) or GBR12909 (5 mg/kg) (WT, filled squares, *n* = 6; and D5R-KO, open squares, *n* = 6). The pretreatments were administered 80 minutes before the METH challenge (arrow). The secondary interaction between blocker pretreatment, genotype, and time course was: F_(11,220)_ = 3.08; and *p*<0.001. The interactions between blocker and genotype at various time points (20, 30, 40, and 50 min after the METH challenge) were F_(1,240)_ = 5.29, 5.63, 4.04, and 6.01; and *p*<0.05at all time points. The main effects of genotype (saline pretreatments) at various time points (10, 20, 30, 40, 50, and 60 min after the METH challenge) were F_(1,240)_ = 4.08, 18.07, 20.08, 15.01, 15.58, and 7.56; and *p* = <0.05, *p*<0.0001, *p*<0.0001, *p*<0.001, *p*<0.001, and *p*<0.01, respectively. The main effects of GBR12909 on D5R-KO mice at various time points (10, 20, and 50 min after the METH challenge) were F_(1,240)_ = 5.37, 7.99, and 4.68; and *p*<0.05×10^−2^, *p*<0.01, and *p*<0.05, respectively. * *p*<0.05 for main effect of genotype; and # *p*<0.05 for main effect of blocker. (b) Open field locomotor activity after challenge with cocaine (15 mg/kg). Ambulation of two experimental groups of mice (*n* = 8 each) after cocaine challenge. Saline injection (arrowhead) was performed 80 minutes before the cocaine challenge (arrow). WT, filled circles; D5R-KO, open circles. The interaction between genotype and challenge was F_(1,154)_ = 1.00 and *p* = 4.49×10^−1^.

The three-way ANOVA found a secondary interaction between the three factors (blocker×genotype×time course) (F_(11, 220)_ = 3.08; and *p*<0.001). In addition, post-hoc analyses found a simple interaction between blocker and genotype at 20, 30, 40, and 50 min after the METH challenge (Figure 2a; F_(1, 240)_ = 5.29, 5.63, 4.04, and 6.01; and *p*<0.05 at all time points). Subsequent post-hoc analyses also found simple main effects of blocker and genotype. The effects of blocker were only significant in D5R-KO mice at 10, 20, and 50 min after the METH challenge (F_(1, 240)_ = 5.37, 7.99, and 4.68; and *p*<0.05, *p*<0.001, and *p*<0.05, respectively). The effects of genotype were only significant at 10, 20, 30, 40, 50, and 60 min after the METH challenge in the saline control pretreated group (F_(1, 240)_ = 4.08, 18.07, 20.08, 15.01, 15.58, and 7.56; and *p*<0.05, *p*<0.0001, *p*<0.0001, *p*<0.001, *p*<0.001, and *p*<0.01, respectively). Consistent with previous studies [14], [21], [22], the METH challenge increased ambulation in D5R-KO mice by approximately 200–260% in animals that were pretreated with saline (Figure 2a). METH -induced ambulation in D5R-KO mice was significantly greater than in WT mice from 0 to 60 min after the METH challenge. At some of the time points, D5R-KO mice traveled a total distance up to 30% greater than that traveled by WT mice. Thus, pretreatment with the DAT blocker specifically eliminated METH-induced hyperlocomotor activity in D5R-KO mice. By contrast, cocaine (15 mg/kg)-induced ambulation was not significantly different between WT and D5R-KO mice (Figure 2b).

### DAT phosphorylation in D5R-KO mice

**Figure 3:**
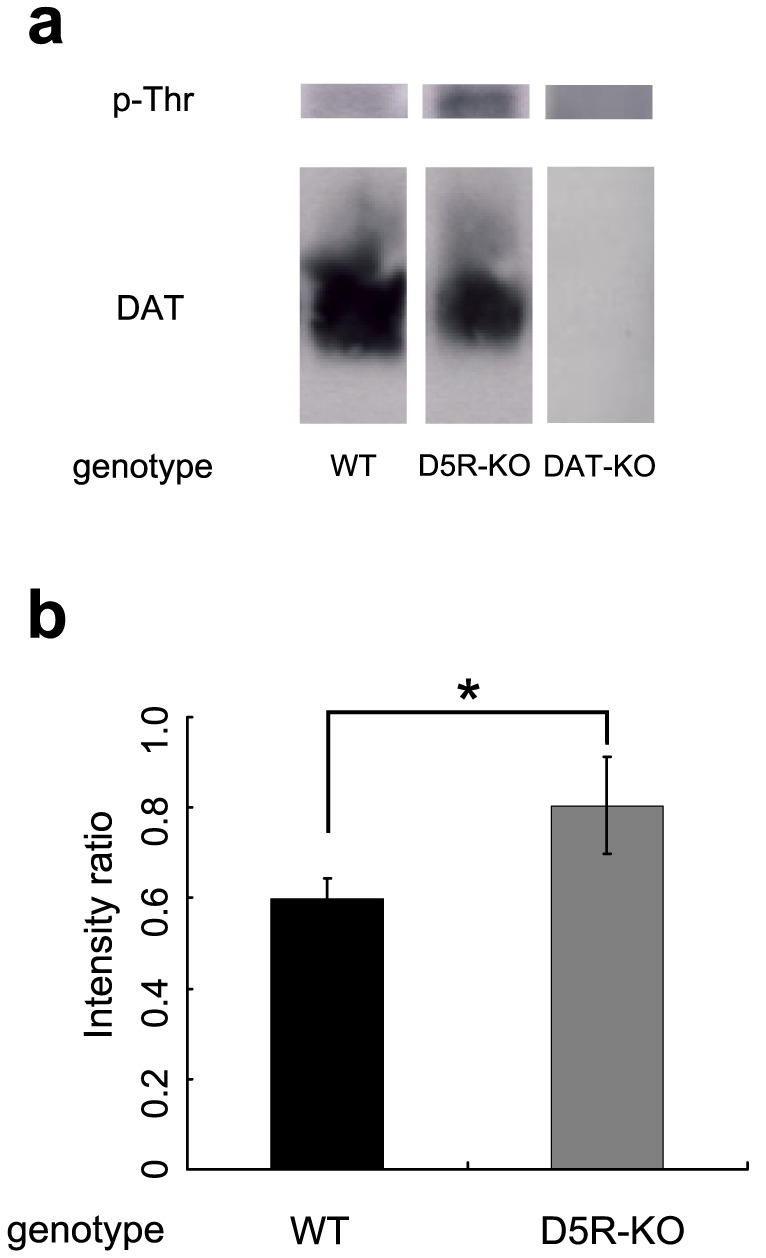
DAT phosphorylation in D5R-KO mice. DAT proteins were immunoprecipitated from whole brain lysates and were then immunoblotted with anti-phosphothreonine and anti-DAT antibodies. (a) Upper: Representative Western blot of phospho-threonine signals of immunoprecipitated DAT proteins. Lower: Total DAT protein levels of the same samples. WT, D5R-KO, and DAT-KO genotypes are indicated. (b) The intensities of the phosphothreonine bands were normalized to the intensities of the total DAT protein bands to quantify the phosphothreonine levels. WT mice, black bar; D5R-KO mice, gray bar. Threonine phosphorylation levels were significantly increased in D5R-KO mouse brains relative to WT mouse brains. Paired t-test: *t* = −2.59 and *p*<0.05. * *p*<0.05.

Because the DAT blocker eliminated METH-induced hyperlocomotor activity in D5R-KO mice and there were no differences in cocaine-induced locomotor activity between D5R-KO and WT mice, we hypothesized that the METH-induced hyperactivity resulted from a change in DAT activity in D5R-KO mice. Previous studies found that DAT activity is regulated by phosphorylation. Phosphorylation of the 53^rd^ threonine residue in the N-terminus of DAT is required for amphetamine-induced neurotransmitter release that is mediated by monoamine transporters [17], [18], [19]. Therefore, we assessed threonine phosphorylation of DAT immunoprecipitates that were made from whole brain lysates. The detection of a signal at 75 kDa by anti-phospho-Thr antibody was DAT(+/+)-dependent since no signal was detected in the immunoprecipitant from DAT-KO mice (Figure 3a). An ANOVA revealed that threonine phosphorylation levels per total DAT protein level were significantly higher in immunoprecipitates made from D5R-KO mouse brain lysates than in immunoprecipitates made from WT mouse brain lysates (Figure 3a, b; *t* = −2.59, *p*<0.05).

### Dopamine transmission in D5R-KO mice

**Figure 4:**
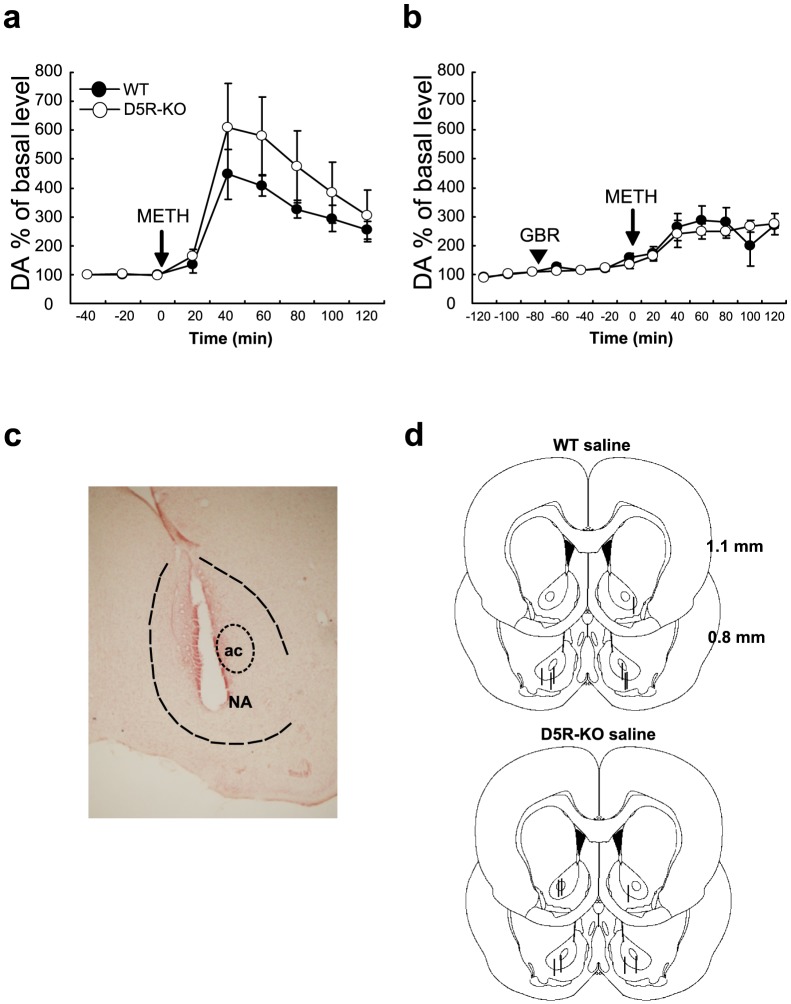
DAT levels in D5R-KO mice. (a) Dialysate samples were collected at a sampling rate of 2 µl/min for 20 min during a baseline period of 60 min and then for an experimental period of 120 min following the METH challenge (2.5 mg/kg; arrow). The interaction between blocker pretreatment and challenge was F_(5,120)_ = 6.66 and *p*<0.0001. The simple main effects of blocker pretreatment at 40 and 60 min were F_(1,144)_ = 13.50 and 8.08, and *p*<0.001 and *p*<0.001, respectively. WT, filled circles (*n* = 7); D5R-KO, open circles (*n* = 7). DA, dopamine. The arrowhead indicates the time point of saline injection. (b) Dialysate samples were collected for 20 min during a baseline period of 60 min. The animals were then injected with GBR12909 (5 mg/kg) and samples were collected for 20 min during a pretreatment period of 80 min. The animals were then challenged with METH (2.5 mg/kg) and samples were collected throughout the 120 min experimental period. The arrowhead indicates the GBR12909 pretreatment and the arrow indicates the METH challenge. (c) Representative microdialysis probe placements. Dashed lines denote the boundaries of the NA and the anterior commissure (ac). (d) Schematic representations of probe placements in the experimental groups for microdialysis of the NA at two different rostrocaudal levels (1.1 mm and 0.8 mm rostral from the bregma). The short lines indicate the probe tracks. Seven WT and seven D5R-KO probe track cases are overlaid on representative sections.

To determine the brain region responsible for the difference in hypersensitivity to METH between WT and D5R-KO mice, we next investigated fluctuations in dopamine levels in the NA. The NA receives inputs from dopamine neurons that regulate locomotor activity [23]. *In vivo* microdialysis was performed in freely moving mice to measure dopamine levels (Figures 4a, b, c, d). Dopamine levels in the NA were increased by approximately 350% in WT and 400% in D5R-KO mice from 20 to 40 minutes after the METH challenge. By 120 minutes after the METH challenge, the dopamine levels in the NA of both genotypes decreased to slightly more than twice the baseline levels (Figure 4a). The dopamine fluctuation ratios relative to the baselines were analyzed for the different pretreatment and genotype groups. A three-way ANOVA (blocker×genotype×time course) and subsequent post-hoc analyses found a significant interaction between blocker and time course after the METH challenge (Figure 4a, b; F_(5, 120)_ = 6.66; and *p*<0.0001). Post-hoc analyses also found a simple main effect of blocker at the 40 and 60 min time points after the METH challenge (Figure 4a, b; F_(1, 144)_ = 13.5 and 8.08; and *p*<0.001 and *p*<0.01, respectively). Dialysis probe locations were confirmed by visual examination of probe track (Figure 4c), and all microdialysis data used for analyses were obtained from the NA (Figure 4d). Thus, dopamine transmission in the NA, which is thought to be required for METH-induced increases in locomotor activity, was blocked by GBR12909. However, the secondary interactions between blocker, genotype, and time course were not significant and there were no differences in dopamine transmission in the NA across genotypes (F_(5, 120)_ = 1.44; and *p* = 2.14×10^−1^). Thus, differences in METH-induced dopamine transmission in the NA do not seem to contribute to the higher METH-induced locomotor activity of D5R-KO mice.

## Discussion

Dopamine signaling in the NA mediates psychostimulant-induced locomotion [24], [25], [26]. In the present study, we found that D5R-KO mice were more sensitive to a METH challenge (2.5 mg/kg) than WT control mice. This greater sensitivity of D5R-KO mice to acute METH administrations was almost completely blocked by pretreatment with the DAT blocker GBR12909 (5 mg/kg). However, pretreatment with GBR12909 did not prevent METH-induced locomotor activity. In addition, our results showed that there were no differences in cocaine-induced locomotor activities between genotypes. These observations are consistent with previous studies, which found that a different line of D5R-KO mice were not hypersensitive to acute cocaine administrations [16]. These observations suggest that alterations in METH-induced dopamine release through the DAT pore likely account for the increased locomotor activity observed in D5R-KO mice. By contrast, other processes such as quantum release-dependent elevations of dopamine levels do not seem to contribute to the enhanced locomotor activity observed in METH-challenged D5R-KO mice. In addition, saline challenge coupled with GBR12909 pretreatment did not cause differences in locomotor activity between genotypes. Furthermore, postsynaptic processes that are induced by synaptic dopamine transmission did not underlie the enhanced locomotor activity observed in D5R-KO mice. Pretreatment with GBR12909, which completely blocks dopamine release through the DAT pore, eliminated the METH-induced increase in locomotor activity in D5R-KO mice. Thus, the DAT pore is required for the observed hypersensitivity to METH in D5R-KO mice. Therefore, one of the crucial consequences of D5R deficiency is alterations in METH-induced DAT activity.

We were unable to determine the precise anatomical targets of D5R deficiency. We did not find differences in the ratios of METH-induced dopamine levels relative to baselines in the NA between genotypes. Thus, the NA is not a direct anatomical target of D5R deficiency. However, neuronal circuits that include dopaminergic terminals are affected by D5R deficiency because DAT blockade specifically eliminated the METH-induced hyperlocomotor activity that was observed in D5R-KO mice. Interestingly, synaptic transmission of norepinephrine in the mPFC influences the dopaminergic signaling in the NA that regulates amphetamine-induced stimulation of ambulation. This influence is likely mediated by direct glutamatergic signaling from the mPFC to the NA [27], [28]. Thus, norepinephrine transmission in the mPFC may also be affected by D5R deficiency.

The phosphothreonine levels of DAT were significantly increased in the brains of D5R-KO mice relative to WT mice. These data indicate that D5R activity regulates the phosphorylation status of DAT, which in turn affects METH-induced dopamine release. Indeed, recent studies demonstrated that threonine 53 in DAT is required for amphetamine-induced dopamine release [17], [18], [19].

Previous studies that utilized a different line of D5R-KO mice found a response to cocaine that we did not observe in the D5R-KO mice described here [15], [16]. Differences in the genetic background of the two mouse lines may explain the discrepancies in the responses to cocaine. D5R-KO mice with a mixed genetic background (hybrid of 129/SvJ1×C57BL/6J) are less sensitive to cocaine than WT mice [15]. By contrast, when this mixed background line of D5R-KO mice is bred onto a C57B6 genetic background, there are no differences in sensitivity to cocaine between D5R-KO and WT mice [16]. Our results were consistent with the findings of the later study. Moreover, other studies that use crossbred rather than genetically engineered mice also find that sensitivity to METH is dependent upon the genetic background of the mice [29], [30], [31]. More specifically, a crossbred line of mice that has an extreme sensitivity to METH also exhibits hypersensitivity to cocaine. Because these results contrast with the genetically engineered D5R-KO line used in our study, it seems likely that the locus responsible for the hypersensitivity to cocaine in the crossbred mice is not the D5R locus. Complimentary experiments that cross our genetically engineered D5R-KO line with F1 generation mice from each of these other lines should be performed to determine if these other lines actually have alterations in the D5R locus.

The clinical relevance of our findings is unclear because METH is an exogenous substance. However, epidemiological studies find a potential relation between D5R deficiency and behavior. For example, a small population of people has microsatellites in the D5R gene locus that may attenuate D5R gene expression [32]. These individuals with a microsatellite polymorphism in the D5R gene locus (148 bp allele) are also at high risk of becoming drug abusers [13], [14].

In summary, the results presented here provide the first evidence of a functional relationship between the D5R gene and DAT. This link between D5R gene functions and DAT activity might help to elucidate more complex multigene dysfunctions that underlie diseases that cannot be attributed to alterations in one gene.

## Methods

### Ethics Statement

The Animal Care and Use Committee of the Tokyo Metropolitan Institute for Neuroscience approved all surgical and experimental protocols that were used in this study (Permit Number: 08-1815).

### Generation of D5R-KO mice

The WT D5R gene consists of a 9.0 kb genomic fragment that contains the receptor coding region and a 9.5 kb genomic fragment that contains the upstream region. The mutant D5R construct “pD5KO” consisted of part of the 9.0 kb coding region genomic fragment and the complete 9.5 kb upstream genomic fragment. The fragments were isolated from a 129/SvJ1 genomic library and subcloned as EcoRV–NheI (9.0 kb) and XbaI–XbaI (9.5 kb) fragments into the same gene targeting vector. The targeting vector was constructed so that the entire coding sequence was deleted by using the following DNA fragments: a 1.2 kb MC1 promoter-diphtheria toxin-A fragment gene (DT-A) for negative selection; the XbaI–XbaI fragment containing the upstream region of the D5R gene; a 2.3 kb PGK promoter-*Escherichia coli* xanthine-guanine phosphoribosyltransferase gene (gpt); a 1.1 kb MC1 promoter-neomycin gene (neo); a 4.0 kb XbaI–NheI fragment containing the 3′-untranslated region; and a pBluescript plasmid (Figure 1a). This targeting vector was then used to generate D1 dopamine receptor-deficient mice [33].

The ES cell line used for generation of the D5R-KO mice was previously described [33]. Briefly, CCE ES cells (E14TG2a IV 129-derived ES cells, acquired from Dr. E. Robertson) were cultured as described [34]. The cultured ES cells (2.5×10^7^ cells) were transfected with the linearized targeting vector (50 µg) by electroporation, and then cells that contained the targeting vector were selected with G418. A total of 120 drug-resistant colonies were collected, and then the genomic DNA was digested with EcoRV and NheI and subjected to Southern blot analysis for confirmation of homologous recombination (Figure 1b). D5R-KO mice were then generated using the homologous recombinant ES cells. The mice were backcrossed with a C57BL/6J strain for 10 generations and were then maintained on the C57BL/6J genetic background. D5R-KO heterozygous males and females with a C57BL/6J genetic background were crossed to generate D5R-KO homozygous lines and WT homozygous lines. All of the mice used in the experiments were generated by matings between D5R-KO homozygous mice and between WT mice.

Tail DNA was analyzed by PCR with three primers: D5R-5-type-in-1, 5′-CGTCCTGGAATTGTGACTCTGCTG-3′; D5R-5P-out-1, 5′-TAGGGGTTTGGGATAAGGTTGTGA-3′; and neo10, 5′-ATCAGAGCAGCCGATTGTCTGTTG-3′. The PCR conditions used for genotyping were denaturation at 94°C for 4 min, followed by 30 cycles of 94°C for 1 min, 1 min at 55°C, and 1 min at 72°C. PCR products were stored at 4°C until use. The WT and mutant alleles resulted in PCR products of 225 and 450 bp, respectively.

Brain mRNA was prepared according to standard protocols and subjected to Northern blot analysis to confirm that the D5R-KO mice did not contain the D5R transcript (Figure 1c).

### Locomotor activity measurements

The horizontal movements of the animals were captured with a CCD camera connected to a computer with a video-capture board. The camera was operated using Linux software (IO Data, Japan), and the animals were tracked with an I-rec webcam system [35]. The travel distances were calculated with custom-made scripts. Mice were familiarized to an open field (40×40 cm) for 1 h. The next day, mice were injected with saline and the locomotor activities were recorded for 1 h. Then, the mice were exposed to either a METH (2.5 mg/kg) or a cocaine challenge (15 mg/kg) and the locomotor activities were recorded for an additional 1 h. For the blocking experiments, either saline or GBR12909 (5 mg/kg) was administered by intraperitoneal injections that occurred 80 min before the METH challenge. Six time points (six time bins of 10 min each) were analyzed after the challenges.

### Microdialysis

Microdialysis probes (type AG; outer diameter: 0.5 mm; inner diameter: 0.4 mm; Eicom, Kyoto, Japan) were stereotaxically implanted under anesthesia to target the NA and were then fixed to the skull. The coordinates used were: anterior-posterior 1.1 mm; medial-lateral 1.2–1.6 mm; and dorsal-ventral 3.8–4.4 mm. Experimental dialysate samples were collected on days 1 and 2 after the surgeries. The microdialysis probes were perfused with artificial cerebrospinal fluid at a flow rate of 2 µl/min and with a sampling time of 20 min.

Once the perfusions began, the mice were then left alone for either 2 h (day 1) or 1 h (day 2) to stabilize the baseline measurements. Immediately following stabilization on day 1, saline was injected and samples were collected during a baseline period of 60 min. Next, METH (2.5 mg/kg) was injected and samples were collected during an experimental period of 120 min. On day 2, mice were pretreated with GBR12909 (5 mg/kg) for 20 and 80 min prior to the saline and METH injections, respectively. Nine and thirteen dialysate samples were obtained from the NA on day 1 (baseline and METH treated) and on day 2 (baseline, GBR12909, and METH treated), respectively. Forty microliters (2 µl/min for 20 min) of dialysates were collected at 4°C in a polypropylene sample tube during each session. The dialysate samples were stored under acidic conditions (pH 3.5). Dialysate samples were also collected from D5R-KO mice that were only treated with GBR12909. All of the samples were immediately frozen and stored at −20°C until use. The dopamine content of the samples was assessed as previously described [36].

### Immunoprecipitations and Western blotting

Whole brain lysates from WT and D5R-KO mouse brains were prepared for Western blotting. For each experimental and control condition, eight hemispheres from a total of six mice that were 12 weeks old were homogenized in ice-cold buffer (50 mM Tris-HCl pH 7.5, 150 mM NaCl, 0.1% sodium dodecyl sulfate (SDS), 1% sodium deoxycholate, 1% Triton, 50 mM NaF, and 2 mM EDTA) that was supplemented with 2 mM Na_3_VO_4_, a phosphatase inhibitor cocktail (Sigma Chemical Company, St. Louis, MO, USA) and protease inhibitors (Roche Diagnostics, Indianapolis, IN, USA). The insoluble proteins were removed by centrifugation at 10,000×g for 10 min. The lysates (supernatants) were then incubated for 4 h with protein G agarose beads (50 µl) at 4°C.

Immunoprecipitations were then performed overnight at 4°C using 1 µl of rat DAT antibody (Chemicon MAB369, Chemicon, Temecula, CA, USA) and 2 mg of protein lysate. The volume of antibody-coated agarose beads used per mg of protein was approximately 0.5 µl. The beads were washed three times with 1 ml of immunoprecipitation buffer. Proteins were eluted by addition of 2×SDS sample buffer (25 µl; 125 mM Tris-HCl pH 6.8, 10% 2-mercaptoethanol, 4% SDS, 10% sucrose, and 0.01% bromophenol blue) and 5 min of boiling. The eluted samples (10 µl) were then separated on 10% SDS-PAGE gels and transferred to polyvinylidene difluoride (PVDF) membranes at 100 V for 1 h. The membranes were exposed to a phosphothreonine primary antibody (Cell Signaling Technology #9381; 1∶1000; Cell Signaling Technology, Beverly, MA, USA) and then a horseradish peroxidase-coupled donkey anti-rabbit IgG secondary antibody (1∶200; GE Healthcare, Waukesha, WI, USA). The bands on the Western blots were visualized with an enhanced chemiluminescence kit (GE Healthcare) and exposure to Hyperfilm ECL (GE Healthcare).

### Statistical analyses

Data were analyzed with the “R” programming environment and software language created by the R Foundation for Statistical Computing (Vienna, Austria). The changes in ambulatory activities between baselines and METH challenges were calculated by subtracting the average ambulation that occurred during the last 30 min of the baseline periods from the average ambulation that occurred during each of the 10 min time bins after the METH challenges. The ambulatory activities after the METH challenges were analyzed using a three-way ANOVA for blocker, genotype, and time (2×2×6). Post-hoc analyses were then used to determine secondary interactions between the three factors. Simple interactions between blocker and genotype were subjected to further post-hoc analyses. Specifically, the simple main effects of blocker (saline or GBR12909) and genotype on locomotor activity were assessed for each 10 min time bin.

Microdialysis data were also analyzed using a three-way ANOVA for blocker, genotype, and time (2×2×6). The post-hoc analyses were performed as described above for the ambulation experiments. DAT phosphorylation data were analyzed using a two-tailed paired *t*-test. Because it was difficult to control for the variance in the background levels across the different Western blot experiments, the data from each experiment were treated as a pair (WT and D5R-KO) in the *t*-test. Data are presented as mean ± s.e.m. throughout the manuscript.

## References

[1] Liebman JM, Butcher LL (1974) Comparative involvement of dopamine and noradrenaline in rate-free self-stimulation in substania nigra, lateral hypothalamus, and mesencephalic central gray. Naunyn Schmiedebergs Arch Pharmacol 284: 167–194. 4152814 10.1007/BF00501121

[2] Yokel RA, Wise RA (1975) Increased lever pressing for amphetamine after pimozide in rats: implications for a dopamine theory of reward. Science 187: 547–549. 1114313 10.1126/science.1114313

[3] Fouriezos G, Wise RA (1976) Pimozide-induced extinction of intracranial self-stimulation: response patterns rule out motor or performance deficits. Brain Res 103: 377–380. 1252926 10.1016/0006-8993(76)90809-x

[4] De Wit H, Wise RA (1977) Blockade of cocaine reinforcement in rats with the dopamine receptor blocker pimozide, but not with the noradrenergic blockers phentolamine or phenoxybenzamine. Can J Psychol 31: 195–203. 608135 10.1037/h0081662

[5] Miller R, Wickens JR, Beninger RJ (1990) Dopamine D-1 and D-2 receptors in relation to reward and performance: a case for the D-1 receptor as a primary site of therapeutic action of neuroleptic drugs. Prog Neurobiol 34: 143–183. 1969668 10.1016/0301-0082(90)90005-2

[6] Berridge KC, Robinson TE (1998) What is the role of dopamine in reward: hedonic impact, reward learning, or incentive salience? Brain Res Brain Res Rev 28: 309–369. 9858756 10.1016/s0165-0173(98)00019-8

[7] Pessiglione M, Seymour B, Flandin G, Dolan RJ, Frith CD (2006) Dopamine-dependent prediction errors underpin reward-seeking behaviour in humans. Nature 442: 1042–1045. 16929307 10.1038/nature05051 PMC2636869

[8] Xu M, Hu XT, Cooper DC, Moratalla R, Graybiel AM, et al (1994) Elimination of cocaine-induced hyperactivity and dopamine-mediated neurophysiological effects in dopamine D1 receptor mutant mice. Cell 79: 945–955. 8001143 10.1016/0092-8674(94)90026-4

[9] Cunningham CL, Howard MA, Gill SJ, Rubinstein M, Low MJ, et al (2000) Ethanol-conditioned place preference is reduced in dopamine D2 receptor-deficient mice. Pharmacol Biochem Behav 67: 693–699. 11166059 10.1016/s0091-3057(00)00414-7

[10] Fadok JP, Dickerson TM, Palmiter RD (2009) Dopamine is necessary for cue-dependent fear conditioning. J Neurosci 29: 11089–11097. 19741115 10.1523/JNEUROSCI.1616-09.2009 PMC2759996

[11] Ouyang M, Young MB, Lestini MM, Schutsky K, Thomas SA (2012) Redundant catecholamine signaling consolidates fear memory via phospholipase C. J Neurosci 32: 1932–1941. 22323706 10.1523/JNEUROSCI.5231-11.2012 PMC3306178

[12] Missale C, Nash SR, Robinson SW, Jaber M, Caron MG (1998) Dopamine receptors: from structure to function. Physiol Rev 78: 189–225. 9457173 10.1152/physrev.1998.78.1.189

[13] Vanyukov MM, Moss HB, Gioio AE, Hughes HB, Kaplan BB, et al (1998) An association between a microsatellite polymorphism at the DRD5 gene and the liability to substance abuse: pilot study. Behav Genet 28: 75–82. 9583233 10.1023/a:1021463722326

[14] Vanyukov MM, Moss HB, Kaplan BB, Kirillova GP, Tarter RE (2000) Antisociality, substance dependence, and the DRD5 gene: a preliminary study. Am J of Med Genet (Neuropsychiatric Genetics) 96: 654–658. 10.1002/1096-8628(20001009)96:5<654::aid-ajmg11>3.0.co;2-y 11054773

[15] Elliot EE, Sibley DR, Katz JL (2003) Locomotor and discriminative-stimulus effects of cocaine in dopamine D5 receptor knockout mice. Psychopharmacology (Berl) 169: 161–168. 12768268 10.1007/s00213-003-1494-y

[16] Karlsson RM, Hefner KR, Sibley DR, Holmes A (2008) Comparison of dopamine D1 and D5 receptor knockout mice for cocaine locomotor sensitization. Psychopharmacology (Berl) 200: 117–127. 18600316 10.1007/s00213-008-1165-0 PMC2586326

[17] Foster JD, Pananusorn B, Vaughan RA (2002) Dopamine transporters are phosphorylated on N-terminal serines in rat striatum. J Biol Chem 277: 25178–25186. 11994276 10.1074/jbc.M200294200

[18] Sucic S, Dallinger S, Zdrazil B, Weissensteiner R, Jørgensen TN, et al (2010) The amino terminus of monoamine transporters is a lever required for the action of amphetamines. J Biol Chem 285: 10924–10938. 20118234 10.1074/jbc.M109.083154 PMC2856298

[19] Foster JD, Yang JW, Moritz AE, Challasivakanaka S, Smith MA, et al (2012) Dopamine transporter phosphorylation site threonine 53 regulates substrate reuptake and amphetamine-stimulated efflux. J Biol Chem 287: 29702–29712. 22722938 10.1074/jbc.M112.367706 PMC3436161

[20] Holmes A, Hollon TR, Gleason TC, Liu Z, Dreiling J, et al (2001) Behavioral characterization of dopamine D5 receptor null mutant mice. Behav Neurosci 115: 1129–1144. 11584926

[21] Kuczenski R, Segal DS (1997) Effects of methylphenidate on extracellular dopamine, serotonin, and norepinephrine: comparison with amphetamine. J Neurochem 68: 2032–2037. 9109529 10.1046/j.1471-4159.1997.68052032.x

[22] Vanderschuren LJMJ, Schmidt ED, De Vries TJ, Van Moorsel CAP, et al (1999) A single exposure to amphetamine is sufficient to Induce long-term behavioral, neuroendocrine, and neurochemical sensitization in rats. J Neurosci 19: 9579–9586. 10531460 10.1523/JNEUROSCI.19-21-09579.1999 PMC6782918

[23] Fallon JH, Moore RY (1978) Catecholamine innervation of the basal forebrain. IV. Topography of the dopamine projection to the basal forebrain and neostriatum. J Comp Neurol 180: 545–580. 659674 10.1002/cne.901800310

[24] Delfs JM, Schreiber L, Kelley AE (1990) Microinjection of cocaine into the nucleus accumbens elicits locomotor activation in the rat. J Neurosci 10: 303–310. 2299396 10.1523/JNEUROSCI.10-01-00303.1990 PMC6570324

[25] Sellings LH, Clarke PB (2006) 6-hydroxydopamine lesions of nucleus accumbens core abolish amphetamine-induced conditioned activity. Synapse 59: 374–377. 16463400 10.1002/syn.20247

[26] Riday TT, Kosofsky BE, Malanga CJ (2012) The rewarding and locomotor-sensitizing effects of repeated cocaine administration are distinct and separable in mice. Neuropharmacology 62: 1858–1866. 22197517 10.1016/j.neuropharm.2011.12.011 PMC3269519

[27] Darracq L, Blanc G, Glowinski J, Tassin JP (1998) Importance of the noradrenaline-dopamine coupling in the locomotor activating effects of D-amphetamine. J Neurosci 18: 2729–2739. 9502830 10.1523/JNEUROSCI.18-07-02729.1998 PMC6793121

[28] Ventura R, Cabib S, Alcaro A, Orsini C, Puglisi-Allegra S (2003) Norepinephrine in the prefrontal cortex is critical for amphetamine-induced reward and mesoaccumbens dopamine release. J Neurosci 23: 1879–1885. 12629192 10.1523/JNEUROSCI.23-05-01879.2003 PMC6741949

[29] Kamens HM, Burkhart-Kasch S, McKinnon CS, Li N, Reed C, et al (2005) Sensitivity to psychostimulants in mice bred for high and low stimulation to methamphetamine. Genes Brain Behav 4: 110–125. 15720407 10.1111/j.1601-183X.2004.00101.x

[30] Torkamanzehi A, Boksa P, Ayoubi M, Fortier ME, Ng Ying Kin NM, et al (2006) Identification of informative strains and provisional QTL mapping of amphetamine (AMPH)-induced locomotion in recombinant congenic strains (RCS) of mice. Behav Genet 36: 903–913. 16710777 10.1007/s10519-006-9078-3

[31] Scibelli AC, McKinnon CS, Reed C, Burkhart-Kasch S, Li N, et al (2011) Selective breeding for magnitude of methamphetamine-induced sensitization alters methamphetamine consumption. Psychopharmacology (Berl) 214: 791–804. 21088960 10.1007/s00213-010-2086-2 PMC3320759

[32] Housley DJ, Nikolas M, Venta PJ, Jernigan KA, Waldman ID, et al (2009) SNP discovery and haplotype analysis in the segmentally duplicated DRD5 coding region. Ann Hum Genet 73: 274–282. 19397556 10.1111/j.1469-1809.2009.00513.x PMC2749463

[33] Tran AH, Uwano T, Kimura T, Hori E, Katsuki M, et al (2008) Dopamine D1 receptor modulates hippocampal representation plasticity to spatial novelty. J Neurosci 28: 13390–13400. 19074012 10.1523/JNEUROSCI.2680-08.2008 PMC6671752

[34] Robertson EJ (1987) Teratocarcinomas and Embryonic Stem Cells: A Practical Approach, Robertson EJ (ed). IRL Press: London, pp. 71–112.

[35] Matsuda K “i_rec: a free software to capture of real time movement.” Systems Neuroscience Group's web site. Available: http://staff.aist.go.jp/k.matsuda/eye/. Accessed 2013 Sep 18.

[36] Watanabe M, Kodama T, Hikosaka K (1997) Increase of extracellular dopamine in primate prefrontal cortex during a working memory task. J Neurophysiol 78: 2795–2798. 9356427 10.1152/jn.1997.78.5.2795

